# Supplementary material for: Wear in multiple network elastomers arises from the continuous accumulation of molecular damage rather than microcrack growth
Source: Sci Adv. 2026 May 1;12(18):eaeb9858. doi: 10.1126/sciadv.aeb9858 (PMC13134626; doi:10.1126/sciadv.aeb9858)
Supplement: Supplementary file 1 — Supplementary Text S1 to S13 Figs. S1 to S12 Table S1 References [file sciadv.aeb9858_sm.pdf]

Science Advances
AAAS

Supplementary Materials for

Wear in multiple network elastomers arises from the continuous accumulation of molecular damage rather than microcrack growth

Ombeline Taisne *et al.*

Corresponding author: Jean Comtet, jean.comtet@espci.fr

Sci. Adv. **12**, eaeb9858 (2026)
DOI: 10.1126/sciadv.aeb9858

This PDF file includes:

Supplementary Text S1 to S13
Figs. S1 to S12
Table S1
References

S1. Materials synthesis and characterization

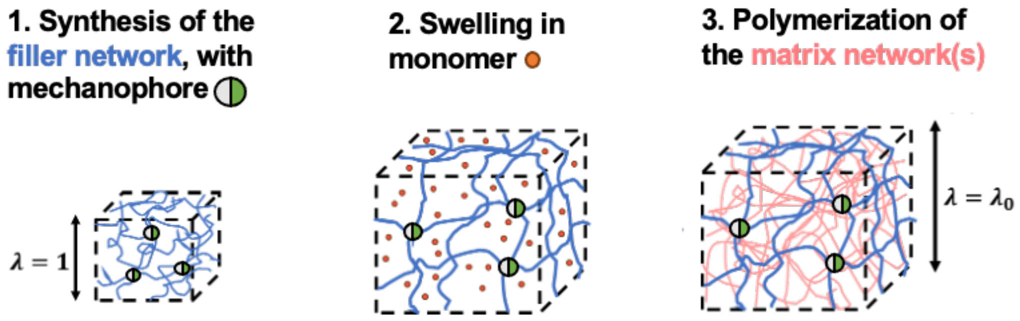

Figure S1: Synthesis of multiple network elastomers, with successive swelling and polymerization steps. Mechanophores are incorporated as part of the cross-linkers in the filler network which is represented in blue. The matrix network is in pink.

A schematic representation of a network with pre-stretch λ_0 is shown in **Figure S1.3**. With the degree of crosslinking used for the filler network, the values of λ_0 measured for a double and a triple network were approximately 1.6 and 2.5 respectively, as controlled by the swelling to equilibrium (see Table 1).

Characteristics of the networks

As described previously in *(47)*, the cross-link density in the filler network, ν_x^{SN}, can be extracted from stress-strain curves of the single network, resulting in $\nu_x^{SN} = 4.2 * 10^{25}\ m^{-3}$. As described in *(46)*, in the case of multiple networks, the cross-link density of the filler network is diluted in the matrix and is given by: $\nu_x^{MN} = \frac{1}{\lambda_0^3} \nu_x^{SN}$.

The final properties of the materials are summarized in Table 1 where the Young's modulus and ν_x^{SN}were taken from *(46,47)*.

Name	ϕ_{filler}	λ_0	[DACL](mol.m^{-3})	E (MPa)	$\nu_x\ (m^{-3})$
**SN**	1	1	2.55	1	$4.2 \cdot 10^{25}$
**DN**	0.22	1.66-1.65	0.56-0.57	1.3	$9.18 - 9.34 \cdot 10^{24}$
**TN**	0.065	2.49	0.17	1.7	$2.7 \cdot 10^{24}$

Table 1: **Summary of the materials.** The double (DNE) and triple (TNE) network elastomers were synthesized from the same single network containing the DACL mechanophore.

S2. Friction setup and calibration

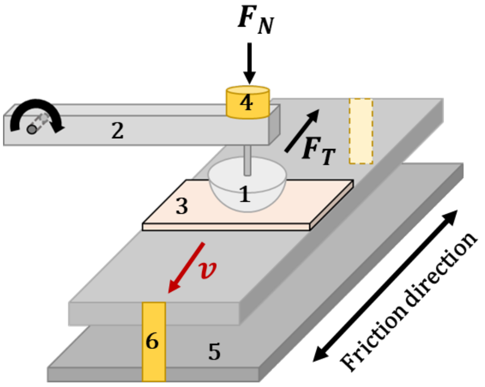

Figure S2: Friction wear setup used for the experiments. *(1)* Glass lens, *(2)* Rotating arm, *(3)* Elastomer, *(4)* Dead load, *(5)* Translation platform, *(6)* Metal strips for tangential force recording.

S3. Characterization of indenter roughness

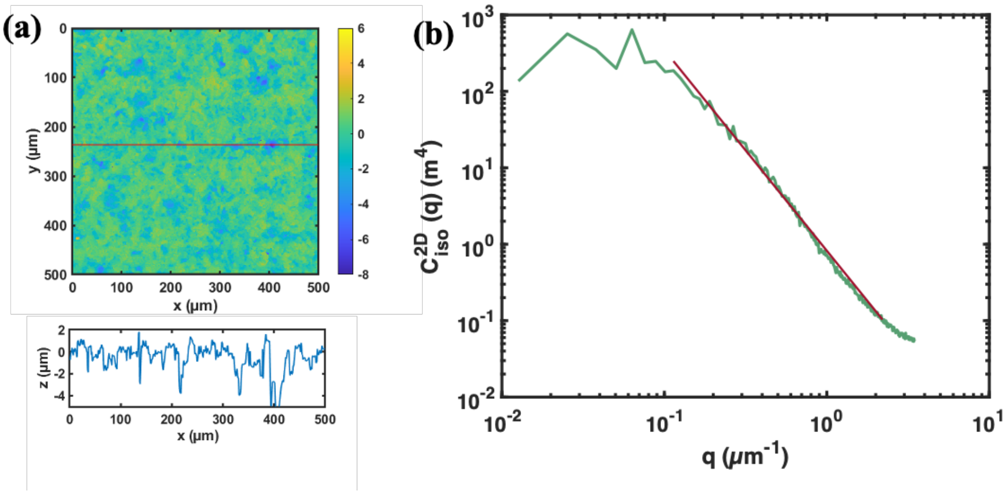

Figure S3: Characterization of the roughness of the indenter (a) Flattened roughness map of the indenter used for all the experiments, the profile below corresponds to the red line. **(b)** $C_{iso}^{2D}(q)$: 2D-radially averaged Power spectral density (PSD) of the roughness map. We fitted the large wave vector region with a power law (red line, $C_{iso}^{2D}(q) \propto q^{-2.6)}$).

The rough indenter used throughout this study was obtained by polishing manually a glass lens (SLB-08-10P from OptoSigma) using p1000 grade sand paper. The surface roughness properties were studied using an optical profilometer (Microsurf 3D from Fogale Nanotech equipped with a x20 objective). 3D roughness maps (500 * 500 µm) were obtained at the apex of the glass lens and were flattened out by fitting the surface with a 4th order polynomial, allowing the extraction of the intrinsic surface roughness. A typical roughness maps and its corresponding surface profile is shown in **Figure S3 (a)**.

Root-mean-square roughness

We used these roughness maps to extract the root mean square (RMS) value of roughness. We obtain a RMS $= 0.99 \pm 0.04$ µm.

Power spectral density

We considered the 2D-radially averaged power spectral density (PSD) $C_{iso}^{2D}(q)$ [μm^4]. The associated PSD profile of the surface is represented in **Figure S3 (b)**, and has a general shape consistent with the literature (80,81). Notably, the PSD is constant for the lower wave vectors in an interval $[q_{min}\ ;\ q_0]$ where $q_{min} = \pi/L$, and q_0 is the so-called "long distance roll-off vector", corresponding to the spatial extension of surface asperities along the x and y directions. Here we have $q_0 \approx 0.139\ \mu m^{-1}$, or $\lambda_0 = 2\pi/q_0 = 45$ µm.

Estimation of the contact area

Using the theory of Bush for asperities occurring on different length scales *(52)*, we can approximate the real area of contact A_r as:

$$A_r = \alpha * F_N = \frac{(1-\nu^2)}{E * \kappa * \sqrt{|\nabla h|^2}} * F_N \qquad (S1)$$

Where $\kappa = \sqrt{2\pi}$, $\nu \approx 0.5$ (Poisson's ratio), E is the Young's modulus of the material (E_{DN} $= 1.3$ MPa, $E_{TN} = 1.7$ MPa) and $|\nabla h|$ can be expressed as a function of the PSD, as *(80)*:

$$\sqrt{|\nabla h|^2} = \left(\frac{1}{2\pi}\int q^3 C^{iso}(q)dq\right)^{\frac{1}{2}} \qquad (S2)$$

Using the PSD spectrum in Figure S3, we obtain $\sqrt{|\nabla h|^2} = 0.84$

The apparent contact area is fixed by Hertz contact theory, where $R = 5.19$ mm is the radius of curvature of the indenter such that:

$$A \approx \left(\frac{9R}{16E}F_N\right)^{\frac{2}{3}} \qquad (S3)$$

Plugging in orders of magnitude, for $F_N \approx 10$ to 100 mN, we find $\frac{A_r}{A} \approx 8$ to 17%, which confirms that real contact occurs on a small portion of the apparent area.

S4. Viscoelastic and adhesive effects on friction

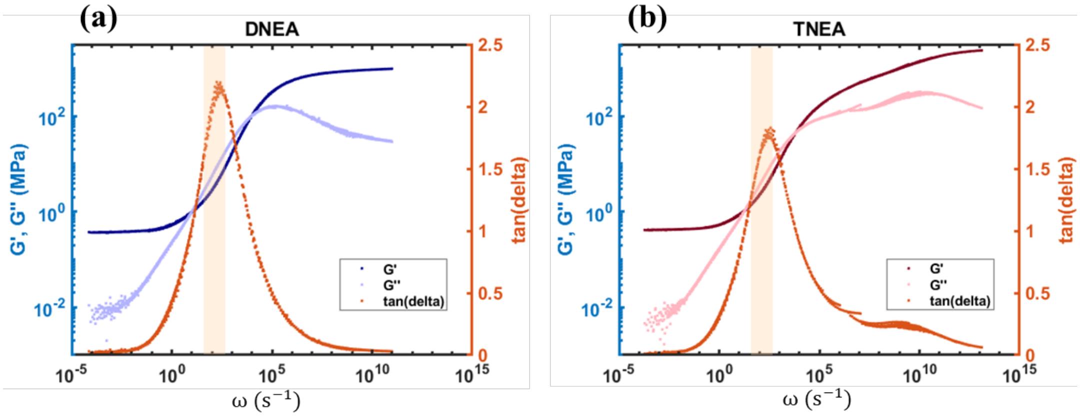

Figure S4: Master curves of the dynamic moduli G'(ω) and G"(ω) and tan (δ) at a reference T of 19°C for DN **(a)** and TN **(b)**. The range of strain rates used in the tribology test ($4.10^1 - 5.10^2 s^{-1}$) is indicated by the orange zone. The rheology data was adapted from (46).

The friction coefficient obtained for DNE and TNE was greater than 1, which can be expected for rubbers due to the potential combination of both visco-elastic and adhesive effects *(82)*.

Viscoelastic effects.

We show in **Figure S4** the evolution of the storage (G') and loss (G'') modulus with frequency, as well as $\tan(\delta) = G''/G'$, the ratio between G'' and G'. Here, we present the data as a master curve, obtained from small strain oscillatory measurements carried out at different temperatures and frequencies. Due to the low sliding speed, we consider that friction should not lead to a significant temperature increase due to heat diffusivity, therefore the reference temperature was taken as the temperature at which the experiment was conducted (19 °C). In our experiments, the average solicitation frequency - or strain rate, can be estimated as: $\dot{\epsilon} = \frac{v}{d}$ with $v = 2\ \mathrm{mm.s^{-1}}$ the sliding speed and d the characteristic length of the contact with an asperity. From the characterization of our indenter roughness, we obtained RMS ≈ 1 µm and a cut-off vector $\lambda_0 \approx 45$ µm (SI.S3). As shown in the paper in **Figure 3A**, the characteristic size of the contact patches is approximately 10 µm. With these considerations, we take a range of $d = 10 - 40$ µm, so that $\dot{\epsilon} \approx 4.10^1 - 5.10^2\ \mathrm{s^{-1}}$. As shown in **Figure S4**, $\dot{\epsilon}$ corresponds to the region of the maximum of tan (δ), corresponding to a state of significant viscoelastic dissipation in the material. Therefore, we can assume that there is a significant contribution of viscoelastic dissipation to the friction force.

Adhesive effects.

We evaluate as well the importance of adhesive effects on the frictional behavior, comparing the elastic energy $E_{\mathrm{el}} = E\lambda h^2$ to fill out the rough cavities through rubber deformation, with the gain in adhesion energy $E_{\mathrm{ad}} = \Delta\gamma\lambda^2$ *(64)*. In these expressions, $E \approx 3 - 30$ GPa is the young modulus taken at the dynamic loading frequency, $h \approx 1$ µm and $\lambda \approx 45$ µm are respectively the characteristic asperity height and in-plane wavelength, taken as the RMS roughness and roll-off wavelength (Fig. S3) and $\Delta\gamma \approx 80\ \mathrm{mJ.m^{-2}}$ the acrylate/glass surface energy [83]. Plugging in these orders of magnitude, we find $E_{\mathrm{ad}}/E_{\mathrm{el}} \approx 0.1 - 1$, showing that adhesion is also expected to play a non-negligible role in the friction force. A similar conclusion is reached when considering other contact adhesion models *(84)*.

S5. Measurement of the abraded volume using optical profilometry

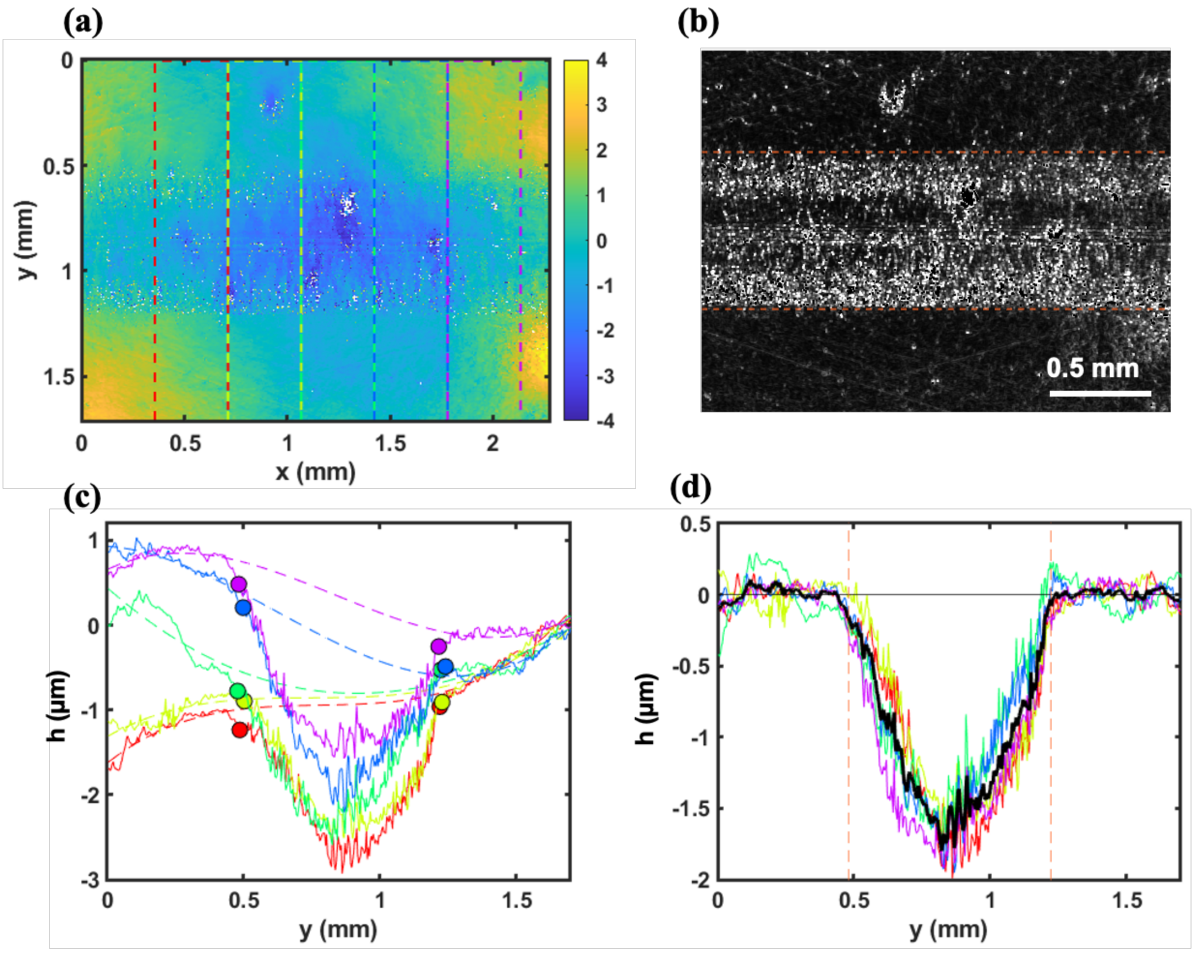

Figure S5: Wear measurement using optical profilometry (DNE, F_N = 42 mN, 5000 cycles) **(a)** Surface topography of a wear mark, color variations account for height fluctuations, in µm, after correcting the tilt of the image (subtraction of a plane). White pixels correspond to non-valid pixels in the image. The image was divided in five zones represented by the dashed boxes. **(b)** 2D Standard deviation of the surface topography, white pixels are zones of higher deviation. The wear mark is delimited by the orange dashed horizontal lines. **(c)** Average height profile taken in the zones delimited by the dashed lines in (a). For each profile, the edges of the wear mark were selected (round markers) and the profile outside these edges was fitted with a 3rd order polynomial (represented here in dashed colored lines). **(d)** Each fit was subtracted from the averaged profiles (colored curves). The averaged, leveled profile over the 5 zones on the wear mark is shown in black. Approximate edges of the wear mark are delimited by the orange dashed lines. The area between the black horizontal line and the profile inside the wear mark is proportional to the volume of worn material.

A typical surface topography is shown in **Figure S5 (a-b)**: after removing the average tilt of the image, the surface of the elastomer showed macroscopic height variations of the order of 1 µm in h for 0.5 mm in y, due to the fabrication of the elastomer. For each image, five average profiles were measured on a different zone of the image. On each profile, the edges of the wear mark were identified, and a profile representative of the pristine surface was obtained by fitting the unworn areas with a 3rd degree polynomial (**Figure S5 c**). For each profile, the area between the unworn and the worn profiles (delimited by the edges) was measured (**Figure S5 d**). The error bars report for the deviation from the average area over the five profiles. One acquisition (2.28 mm length) was taken for each wear mark (7 mm total length) and was positioned in the center of the wear mark in the x-direction. The deviation reported here was of the same order of magnitude as the deviation from one zone of acquisition of the wear mark to another

(excluding the beginning and end of the wear mark in the x-direction). On DNE, for $N < 1000$ cycles, macroscopic wear became harder to detect using optical profilometry. When the abrasion of material from the surface did not lead to a significant difference in roughness between the unworn surface and the worn surface (for example, for $N < 500$ at $F_{\mathrm{N}} = 15$ mN), it was not possible to detect the edges of the wear mark and the worn area was set to zero. Due to the irregularity of the pristine sample, height differences of less than 0.2 μm were hard to detect. For wear marks of approximately 1 mm width, abraded areas of less than 200 μm^2 were within the error of our measurement.

S6. Rescaling of the abraded volume as an Archard wear law

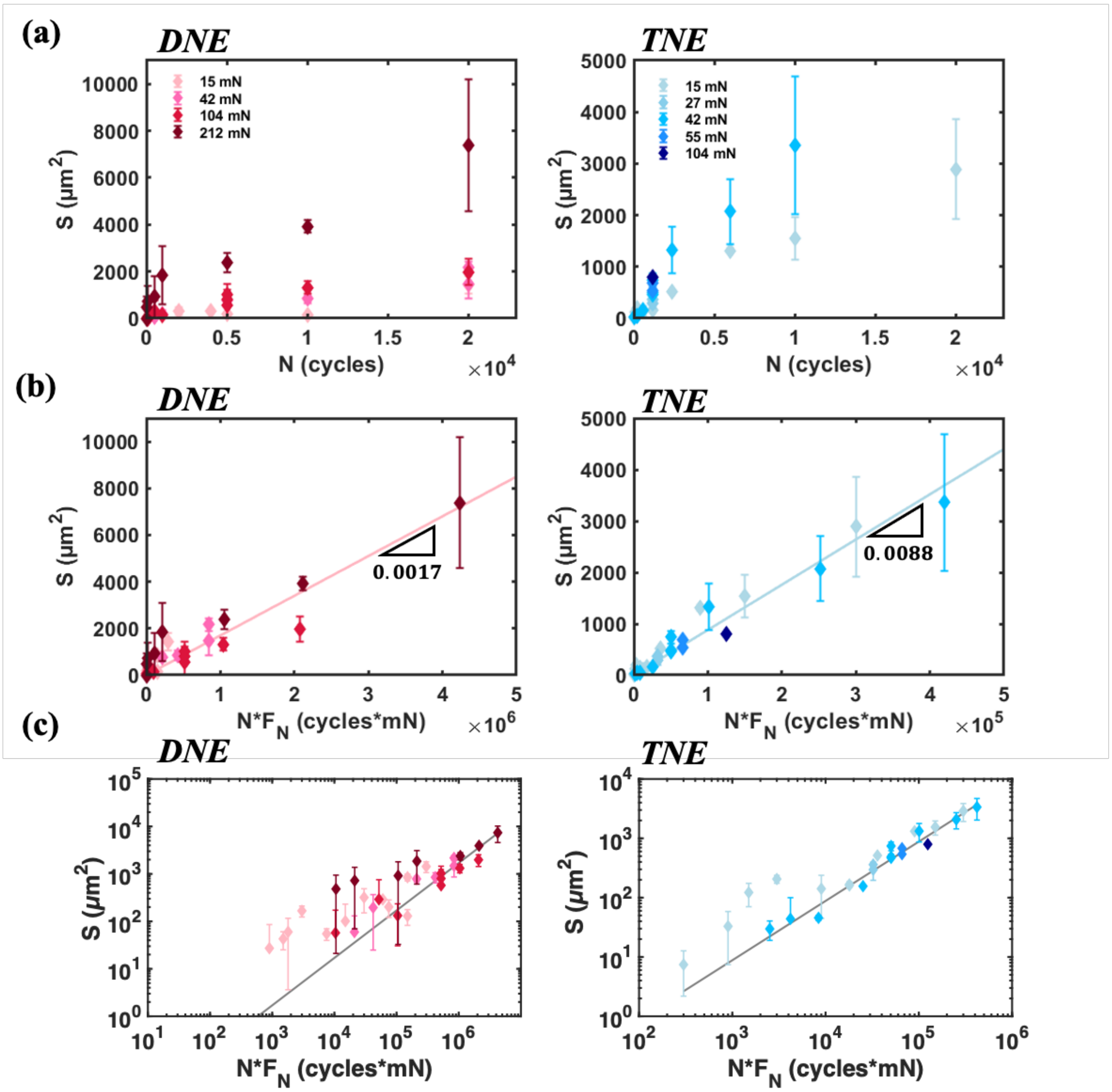

Figure S6: Effect of number of friction cycles and normal force on the abraded volume. (a) Evolution of the abraded area with the number of cycles, depending on the normal force (indicated by different colors) on DNE and TNE. **(b-c)** Rescaled abraded area as a function of $F_N \cdot N$ (normal force times the sliding distance). The lines are linear fits of the experimental data. Error bars show the deviation of the area obtained on 5 zones of the wear mark. Coefficients of linear fits are 0.0017 and 0.0088 $\mu m^2.mN^{-1}.cycle^{-1}$, for DNE and TNE respectively, leading to $k_{DN} = 0.9.10^{-6}$ and $k_{TN} = 6.10^{-6}$ writing $V \approx k.A_r.l$ (see main text for details).

S7. Abraded volume and onset of erosion

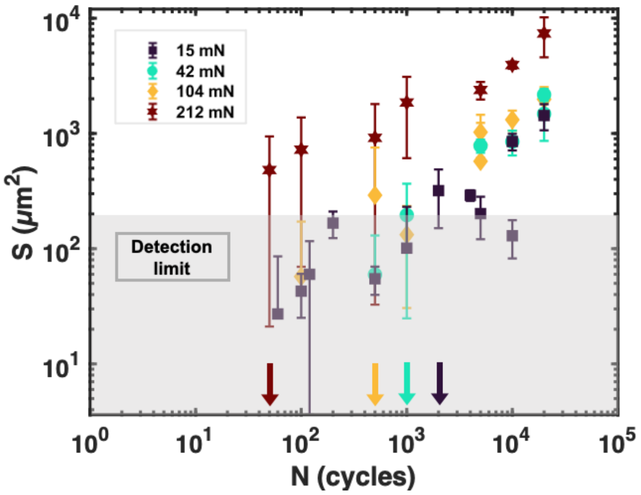

Figure S7: Worn area as a function of number of cycles measured using optical profilometry as described in Figure S5. Error bars show the deviation of the area obtained on 5 zones of the wear mark. The arrows indicate the onset of erosion in the color of the corresponding normal force.

As shown on **Figure S7**, the gray area corresponds to uncertainty in the detection of abraded cross-section, approximately 200 µm^2. We define the onset of the erosion as the number of cycles for which the cross-sectional area *S* overcomes this detection limit. The detection limit is set by the intrinsic irregularity of the elastomer surface, as described in S6.

S8. Confocal imaging and conversion of fluorescence intensity to a fraction ϕ of activated mechanophores

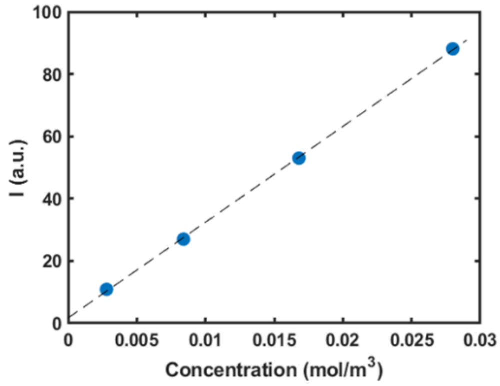

Figure S8: Calibration curve showing the linear relation between fluorescence intensity and concentration of a calibration molecule.

As described previously in *(47)*, to convert fluorescence intensity to a concentration of activated molecules, we used calibration samples. Such calibration samples were prepared by mixing a commercial solution of poly (methyl acrylate) (in toluene) with the calibration molecule: [9-((4-anisyl)ethynyl)anthracene] (home-made synthesis according to the protocol in *(59)*). A drop of this solution was deposited on a coverslip and toluene was evaporated under vacuum overnight, leading to homogeneous samples with a known concentration of calibration molecule. The π-extended anthracene moiety is responsible for the fluorescence observed in the activated part of the mechanophore and in the calibration molecule, and both molecules are assumed to have the same fluorescence properties in the similar environment composed of PMA chains. Therefore, varying the amount of calibration molecule in the calibration samples lead to a calibration curve of fluorescence intensity versus concentration of activated mechanophore, shown in **Figure S8**. The calibration coefficient, α, was extracted from this calibration curve such that $I = \alpha * c + I_{\text{background}}$, with I the fluorescence intensity and c the concentration of calibration molecule.

The concentration of activated mechanophore, $c_{\text{activated}}$ writes as:

$$c_{\text{activated}} = \frac{I}{\alpha} \qquad \text{(S4)}$$

The fraction of activated mechanophores ϕ – which is equal to the fraction of broken chains, then writes as:

$$\phi = \frac{c_{\text{activated}}}{c_0} \qquad \text{(S5)}$$

With c_0 $[\text{mol.m}^{-3}]$, the initial concentration of mechanophore in the material (c_0 depends on the initial pre-strech λ_0, see Table 1, section S1).

Using the image stacks obtained with confocal microscopy, the average in-depth intensity profile $I(z)$ can be obtained. Given the calibration procedure, which is detailed above, the corresponding damage profile can be obtained: $\phi(z) = \frac{c_{\text{activated}}(z)}{c_0}$.

S9. Optical limitations: spatial detection of the worn surface

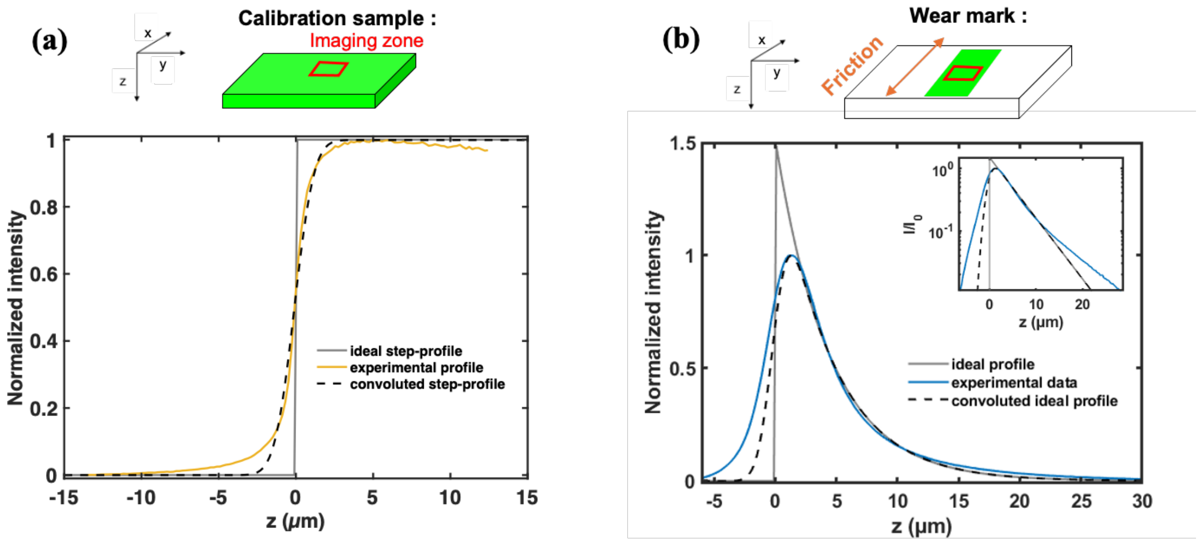

Figure S9: Surface detection with the confocal microscope. (a) Detection of a step profile. Top: schematic representation of a calibration sample. Below: normalized intensity profile measured on a planar interface between (non-fluorescent) glass and a fluorescent calibration sample. Gray line: expected intensity profile characterized by a step function. Due to the non-zero optical section of the objective, this step appears experimentally as a smoother profile. The dashed line represents a step function convoluted with a Gaussian function of variance $\sigma = 1$ μm. **(b) Detection of an intensity profile.** Top: schematic representation of a fluorescent wear mark. Below: Detection of the surface of a fluorescent wear mark. The gray curve corresponds to an ideal intensity profile with an intensity decreasing exponentially below the surface ($I \propto e^{-z/\lambda}$ with here $\lambda = 4.4$ μm). The dashed black curve corresponds to the convolution of the ideal gray profile with a Gaussian function ($\sigma = 1$ μm). The blue curve corresponds to an experimental intensity profile inside a wear mark (TN, $F_N = 15$ mN, 4000 cycles). The inset shows the corresponding profiles in lin-log scale.

The activation profiles in the wear marks were characterized by a progressive increase in intensity upon entry in the material as well as a rounded profile at the intensity maximum. The wear marks were cleaned with ethanol before imaging to get rid of the lubricating layer at the surface. We thus show in the following that these smooth intensity variations at the surface of the wear mark are due to intrinsic limitations associated to the confocal setup.

We first investigate the effect of our imaging system on the detection of a step profile in fluorescence intensity using calibration samples. As shown on **Figure S9 a**, the experimentally measured profile deviates from an ideal step profile. This deviation can be expected due to the finite optical sectioning of the objective. The intensity increase observed experimentally for $z < 0$, and the smoothened profile around $z = 0$ can therefore be attributed to the intrinsic limitations of our optical setup.

To experimentally determine the numerical aperture associated with the objective, we fit the experimental step profile with a gaussian convoluted step function. Picking a variance $\sigma = 1$ μm in **Figure S9 a** allows to describe relatively well the right part ($z > 0$ μm) of the experimentally measured profile but not the part in $z < 0$ μm. In the following, we kept $\sigma = 1$ μm for the sake of simplicity but the discrepancy with the experimental profile suggests that the Point Spread Function (PSF) characterizing the response of our optical system to a step-profile, is more complicated than a simple Gaussian function.

In a second step, we compared in **Figure S9 b**, the intensity profiles obtained experimentally in a wear mark with an idealized profile of maximum intensity at the surface ($z = 0$) decreasing exponentially with depth. We compared the gaussian convolution of this idealized profile with our experimental profile, normalized by the maximum intensity. As was the case with the step-profile (**Figure S9 a**), the experimental profile in **Figure S9 b** increases more smoothly compared to the idealized, convoluted profile, due to our idealized assumption of a purely gaussian PSF. Hence, the convolution of the signal prevents us from distinguishing a profile showing a maximum of intensity at the surface, from one with a maximum less than 1 µm below the surface. Due to this limitation, we define the surface, at z = 0, as the maximum of the intensity profile. Nonetheless this experimental limitation does not preclude us from measurement of the profile in the bulk, since as shown in **Figure S9 b**, the decrease of the intensity gradient is well captured by the convoluted exponential profile.

S10. Comparison of fluorescence activation compared to a pristine surface

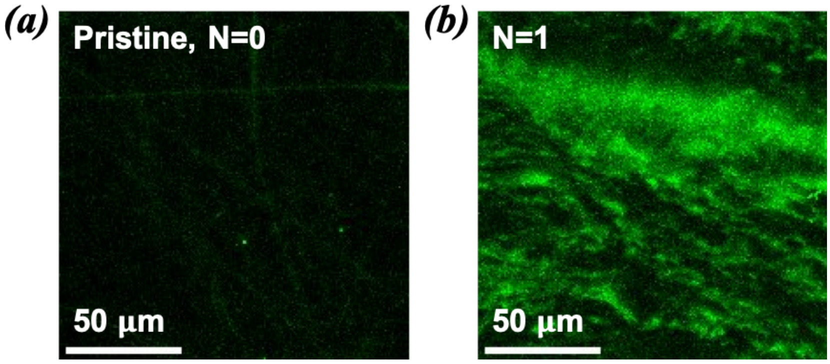

Figure S10: Appearance of molecular damage after a single friction cycle. Maximum intensity projection ***(a)*** at the surface of a pristine DNE sample ($N = 0$), ***(b)*** at the surface of the DNE after a single friction cycle ($N = 1$, $F_N = 42$ mN), where fluorescence patches are observed due to mechanophore activation. Imaging conditions were identical for the two images, which are shown here with the same contrast adjustment. Panel *(b)* is shown in duplicate in Fig. 3 of the main text.

S11. Reporting molecular damage

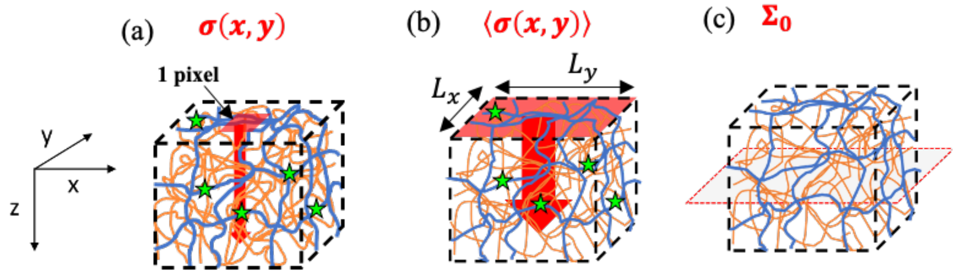

Figure S11: Schematic representation of a multiple network, **(a)** $\sigma(x, y)$ is the local areal density of broken chains in the x-y plane, with the elementary area represented by the red plane (1 pixel), and scission events represented by green stars, **(b)** $\langle\sigma(x, y)\rangle$ is the average areal density of broken chains in the x-y plane, averaged over the area represented by the red plane (size $L_x * L_y$) **(c)** Σ_0 is the areal density of chains in a molecular plane, represented by the dashed red contour.

Surface-averaged fraction of broken chains $\sigma(x, y)$

For damage characterization, we consider $\sigma(x, y)$, the number of broken chains averaged per unit surface area at a specific position (x, y). The unit area is here equal to a single pixel, of size 758*758 nm^2. The surface area which we consider here is in the *x-y* direction, parallel to the surface of the wear mark (see **Figure S11a**, red rectangle). The damage profiles obtained using confocal microscopy give $\phi(x, y, z)$, the fraction of broken chains in the depth of the material. $\sigma(x, y)$ is proportional to the area under the curve of $\phi(x, y, z)$ such that:

$$\sigma(x, y) = \nu_x \int \phi(x, y, z) dz \qquad (S6)$$

With ν_{x}, the volume density of chains between cross-link points, which depends on the pre-stretch of the network (see SI S1, Table 1). The values of $\sigma(x, y)$ which we obtain are of the order of 0.1-1 strands.nm^{-2}.

This local damage value can be averaged over the entire plane of size L_{x}; L_{y}, defining $\langle\sigma\rangle_{\mathrm{x,y}}$ as

$$\langle\sigma\rangle_{\mathrm{x,y}} = \frac{1}{L_{\mathrm{x}} L_{\mathrm{y}}} \iint_{x,y} \sigma(x, y) dx dy \qquad (S7)$$

Areal density of chains Σ_0

From Gaussian statistics, we can express Σ_0^{SN}, the areal density of polymer strands crossing an arbitrary plane in the Single Network material, as *(48)*:

$$\Sigma_0^{\mathrm{SN}} = \frac{1}{2} \cdot \nu_{\mathrm{x}} \langle \mathrm{R}_0^2 \rangle^{\frac{1}{2}} = \frac{\mathrm{l}_0 \mathrm{E}_{\mathrm{x}} \sqrt{\mathrm{C}_\infty \mathrm{N}_{\mathrm{x}}}}{6 \mathrm{k}_{\mathrm{B}} \mathrm{T}} = \mathrm{l}_0 \left(\frac{\mathrm{E}_{\mathrm{x}} \rho \mathrm{N}_{\mathrm{A}} \mathrm{C}_\infty}{6 \mathrm{M}_0 \mathrm{k}_{\mathrm{B}} \mathrm{T}} \right)^{\frac{1}{2}} \qquad (S8)$$

With $\langle R_0^2 \rangle^{\frac{1}{2}}$ the average distance between crosslinks, l_0 the length of a C-C bond, E_{x} the cross-linker contribution to the Young's modulus, ρ the density of the monomer, and M_0 the molar mass of the monomer, T the temperature, and k_{B} the Boltzman constant.

In the case of multiple networks, the areal density of chains of the filler network is diluted in the matrix and is given by: $\Sigma_0^{\mathrm{MNE}} = \frac{1}{\lambda_0^2}\Sigma_0^{\mathrm{SNE}}$

A schematic representation of a single molecular plane passing through the polymer network is given in **Figure S11b**.

Using the λ_0 values determined in SI 1, Table 1, we obtained $\Sigma_0^{\mathrm{DNE}} = 8 \cdot 10^{16}\ \mathrm{m}^{-2}$ for double networks, and $\Sigma_0^{\mathrm{TNE}} = 2.9 \cdot 10^{16}\ \mathrm{m}^{-2}$ for triple networks.

Spatially averaged damage quantity $\bar{\Sigma}$

In our experiments, we consider a normalized value, $\bar{\Sigma}$, given by:

$$\bar{\Sigma} = \frac{\langle\sigma\rangle_{\mathrm{x,y}}}{\Sigma_0} \qquad \text{(S9)}$$

This spatially averaged damage quantity allows us to probe an average damage value, irrespective of the spatial distribution of the intensity profiles. $\bar{\Sigma}$ can also be seen as a number of broken monolayers of meshes in the network, with values ranging from 10 to 100 monolayers in our experiments.

S12. Characterization of the smearing layer

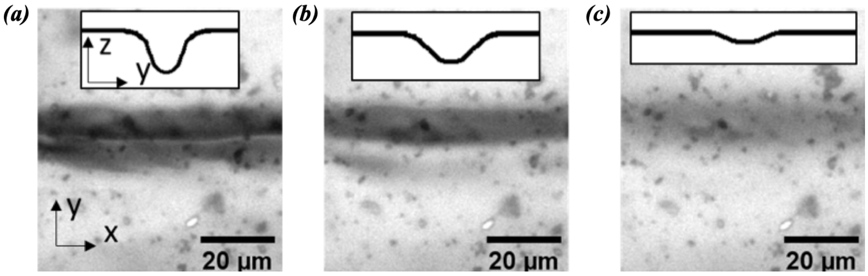

Figure S12: Demonstration of the liquid nature of the third body. Optical microscope images showing the slow relaxation of a groove done in the third body with a needle. ***(a)*** initial groove (central dark horizontal line), ***(b)*** Same groove after 20 s, ***(c)*** After 120 s the groove almost completely fades away.

In order to obtain a coarse estimation of the mechanical properties of the smearing layer, we disturbed its surface with a needle to induce a perturbation of its free surface under the form of a linear groove. As shown in **Figure S12**, this perturbation relaxes with a characteristic time $\tau \approx 100$ s, demonstrating the liquid nature of the smearing layer.

The characteristic time for the groove relaxation can be taken as visco-capillary timescale:

$$\tau \approx \eta R / \gamma \qquad \text{(S10)}$$

With η [Pa.s] the liquid viscosity, γ [N.m^{-1}] the surface tension and R [m] a characteristic distance taken as the groove lateral dimension. Taking $\tau = 100$ s, $\gamma_{\text{ethyl acrylate}} = 26$ mN. m^{-1} *(85)* and $R = 7$ µm, we estimate a dynamic viscosity, $\eta = 4 * 10^5$ Pa. s. This viscosity was comparable to that of a PEA melt of $M_n \approx 60$ kg. mol^{-1} *(86)*.

Using a rough estimation of the end-to-end distance of a PEA chain of $M_n \approx 60$ kg. mol^{-1}, we can evaluate the characteristic size of these chains to be:

$$\sqrt{< R^2 >} \approx a\sqrt{N * C_\infty} \qquad \text{(S11)}$$

Taking $N \approx 600$ monomers, $C_\infty = 9.3$ *(47)* and $a \approx 0.45$ nm (three C-C bonds per monomer, taking $l_{CC} = 0.154$ nm [47]), we obtain : $\sqrt{< R^2 >} \approx 34$ nm.

S13. Weak dependence of damage on the normal force in the erosion regime.

As discussed in Figure 1 of the main text, we can express the erosion speed v_e, assuming that the abraded volume V varies as an Archard law, the abraded surface S will evolve as $S = k.A_\mathrm{R}.N$

We can write $S = 2.h.a_\mathrm{H}$ with $a_\mathrm{H} \sim \left(\frac{3F_\mathrm{N}R}{4E}\right)^{1/3}$ the typical hertz contact radius. This scaling implies that:

$$v_\mathrm{e} = \frac{dh}{dN_\mathrm{cycle}} = k.\frac{A_r}{2.a_\mathrm{H}} = \pi.a_\mathrm{H}.\frac{\mathrm{A_r}}{\mathrm{A}} \qquad \text{(S12)}$$

As detailed in the main text, the damage gradient can be expressed as

$$\phi(z) = (A_r/A)\cdot(1/v_e)\int_z^\infty f(z)dz \qquad \text{(S13)}$$

Reinjecting v_e in this expression leads to

$$\phi(z) = 1/(\pi.a_\mathrm{H})\int_z^\infty f(z)dz \qquad \text{(S14)}$$

i.e. that the erosion and accumulation rate compensate almost exactly, up to a small geometrical prefactor a_H depending weakly on the contact pressure as $a_\mathrm{H} \sim F_N^{1/3}$. This general prediction is in coarse agreement with the experimental observations reported in Figure 4A of a surface damage in steady-state relatively independent of the normal force F_N.

REFERENCES

1. A. I. Vakis, V. A. Yastrebov, J. Scheibert, L. Nicola, D. Dini, C. Minfray, A. Almqvist, M. Paggi, S. Lee, G. Limbert, J. F. Molinari, G. Anciaux, R. Aghababaei, S. Echeverri Restrepo, A. Papangelo, A. Cammarata, P. Nicolini, C. Putignano, G. Carbone, S. Stupkiewicz, J. Lengiewicz, G. Costagliola, F. Bosia, R. Guarino, N. M. Pugno, M. H. Müser, M. Ciavarella, Modeling and simulation in tribology across scales: An overview. *Tribol. Int.* **125**, 169–199 (2018).

2. T. Baumberger, C. Caroli, Solid friction from stick-slip down to pinning and aging. *Adv. Phys.* **55**, 279–348 (2006).

3. B. N. J. Persson, *Sliding Friction: Physical Principles and Applications* (Springer, 2000).

4. H. Bhaskaran, B. Gotsmann, A. Sebastian, U. Drechsler, M. A. Lantz, M. Despont, P. Jaroenapibal, R. W. Carpick, Y. Chen, K. Sridharan, Ultralow nanoscale wear through atom-by-atom attrition in silicon-containing diamond-like carbon. *Nat. Nanotechnol.* **5**, 181–185 (2010).

5. N. Fillot, I. Iordanoff, Y. Berthier, Wear modeling and the third body concept. *Wear* **262**, 949–957 (2007).

6. N. N. Gosvami, J. A. Bares, F. Mangolini, A. R. Konicek, D. G. Yablon, R. W. Carpick, Mechanisms of antiwear tribofilm growth revealed in situ by single-asperity sliding contacts. *Science* **348**, 102–106 (2015).

7. M. Godet, Third-bodies in tribology. *Wear* **136**, 29–45 (1990).

8. V. Vahdat, D. S. Grierson, K. T. Turner, R. W. Carpick, Mechanics of interaction and atomic-scale wear of amplitude modulation atomic force microscopy probes. *ACS Nano* **7**, 3221–3235 (2013).

9. E. Gnecco, R. Bennewitz, E. Meyer, Abrasive wear on the atomic scale. *Phys. Rev. Lett.* **88**, 4 (2002).

10. B. Gotsmann, M. A. Lantz, Atomistic wear in a single asperity sliding contact. *Phys. Rev. Lett.* **101**, 1–4 (2008).

11. K. H. Chung, D. E. Kim, Fundamental investigation of micro wear rate using an atomic force microscope. *Tribol. Lett.* **15**, 135–144 (2003).

12. T. D. B. Jacobs, R. W. Carpick, Nanoscale wear as a stress-assisted chemical reaction. *Nat. Nanotechnol.* **8**, 108–112 (2013).

13. T. Sato, T. Ishida, L. Jalabert, H. Fujita, Real-time transmission electron microscope observation of nanofriction at a single Ag asperity. *Nanotechnology* **23**, 505701 (2012).

14. R. Aghababaei, D. H. Warner, J.-F. Molinari, Critical length scale controls adhesive wear mechanisms. *Nat. Commun.* **7**, 11816 (2016).

15. R. Aghababaei, D. H. Warner, J. F. Molinari, On the debris-level origins of adhesive wear. *Proc. Natl. Acad. Sci. U.S.A.* **114**, 7935–7940 (2017).

16. R. Aghababaei, T. Brink, J. F. Molinari, Asperity-level origins of transition from mild to severe wear. *Phys. Rev. Lett.* **120**, 186105 (2018).

17. B. N. J. Persson, E. Tosatti, Qualitative theory of rubber friction and wear. *J. Chem. Phys.* **112**, 2021–2029 (2000).

18. S. Wagner, T. Hüffer, P. Klöckner, M. Wehrhahn, T. Hofmann, T. Reemtsma, Tire wear particles in the aquatic environment—A review on generation, analysis, occurrence, fate and effects. *Water Res.* **139** 83–100 (2018).

19. A. H. Muhr, A. D. Roberts, Rubber abrasion and wear. *Wear* **158**, 213–228 (1992).

20. K. A. Grosch, A. Schallamach, Relation between abrasion and strength of rubber. *Rubber Chem. Technol.* **39**, 287–305 (1966).

21. K. A. Grosch, Rubber abrasion and tire wear. *Rubber Chem. Technol.* **81**, 470–505 (2008).

22. D. F. Moore, Friction and wear in rubbers and tyres. *Wear* **61**, 273–282 (1980).

23. R. Xu, W. Sheng, F. Zhou, B. N. J. Persson, *Rubber Wear: History, Mechanisms, and Perspectives* (Springer, 2025).

24. H. Liang, Y. Fukahori, A. G. Thomas, J. J. C. Busfield, Rubber abrasion at steady state. *Wear* **266**, 288–296 (2009).

25. M. Huang, M. Guibert, J. Thévenet, C. Fayolle, T. Chaussée, L. Guy, L. Vanel, J. L. Loubet, P. Sotta, A new test method to simulate low-severity wear conditions experienced by rubber tire materials. *Wear* **410–411** 72–82 (2018).

26. Y. Fukahori, H. Yamazaki, Mechanism of rubber abrasion. Part 2. General rule in abrasion pattern formation in rubber-like materials. *Wear* **178**, 109–116 (1994).

27. T. Iwai, Y. Uchiyama, K. Shimosaka, K. Takase, Study on the formation of periodic ridges on the rubber surface by friction and wear monitoring. *Wear* **259**, 669–675 (2005).

28. A. Schallamach, Friction and abrasion of rubber. *Rubber Chem. Technol.* **31**, 982–1014 (1958).

29. S. M. Aharoni, Wear of polymers by roll-formation. *Wear* **25**, 309–327 (1973).

30. M. X. Shen, F. Dong, Z. X. Zhang, X. K. Meng, X. D. Peng, Effect of abrasive size on friction and wear characteristics of nitrile butadiene rubber (NBR) in two-body abrasion. *Tribol. Int.* **103** 1–11 (2016).

31. A. Tiwari, N. Miyashita, B. N. J. Persson, Rubber wear and the role of transfer films on rubber friction on hard rough substrates. *Tribol. Lett.* **69**, 1–12 (2021).

32. A. Schallamach, Abrasion, fatigue, and smearing of rubber. *J. Appl. Polym. Sci.* **12**, 281–293 (1968).

33. A. N. Gent, C. T. R. Pulford, Mechanisms of rubber abrasion. *J. Appl. Polym. Sci.* **28**, 943–960 (1983).

34. K. Daigne, G. Mollon, S. Descartes, N. Fillot, R. Jeanneret-Dit-Grosjean, F. Biesse, A. Perriot, The tire-road contact: A mechanical mixing seen as a shear-induced diffusive process. *Wear* **546–547**, 205339 (2024).

35. K. A. Grosch, Abrasion of rubber and its relation to tire wear. *Rubber Chem. Technol.* **65**, 78–106 (1992).

36. K. A. Grosch, The rolling resistance, wear and traction properties of tread compounds. *Rubber Chem. Technol.* **69**, 495–568 (1996).

37. D. H. Champ, E. Southern, A. G. Thomas, "Fracture mechanics applied to rubber abrasion" in vol. 5 of *Advances in Polymer Friction and Wear* (Springer, 1974), pp. 133–144.

38. E. Southern, A. G. Thomas, Studies of rubber abrasion. *Rubber Chem. Technol.* **52**, 1008–1018 (1979).

39. B. N. J. Persson, R. Xu, N. Miyashita, Rubber wear: Experiment and theory. *J. Chem. Phys.* **162**, 074704 (2025).

40. B. N. J. Persson, Theory of powdery rubber wear. *J. Phys. Condens. Matter* **21**, 485001 (2009).

41. M. Ciavarella, Some remarks on a recent wear theory. *Tribol. Lett.* **73**, 1–8 (2025).

42. M. Ciavarella, Wear due to fatigue initiation. *Tribol. Int.* **207**, 110592 (2025).

43. I. G. Goryacheva, F. I. Stepanov, E. V. Torskaya, Fatigue wear modeling of elastomers. *Phys. Mesomech.* **22**, 65–72 (2019).

44. A. Sakhnevych, A. Genovese, Tyre wear model: A fusion of rubber viscoelasticity, road roughness, and thermodynamic state. *Wear* **542-543** 205291 (2024).

45. E. Ducrot, Y. Chen, M. Bulters, R. P. Sijbesma, C. Creton, Toughening elastomers with sacrificial bonds and watching them break. *Science* **344**, 186–189 (2014).

46. J. Slootman, C. J. Yeh, P. Millereau, J. Comtet, C. Creton, A molecular interpretation of the toughness of multiple network elastomers at high temperature. *Proc. Natl. Acad. Sci. U.S.A.* **119**, 1–11 (2022).

47. J. Slootman, V. Waltz, C. J. Yeh, C. Baumann, R. Göstl, J. Comtet, C. Creton, Quantifying rate- and temperature-dependent molecular damage in elastomer fracture. *Phys. Rev. X* **10**, 041045 2020.

48. P. Millereau, E. Ducrot, J. M. Clough, M. E. Wiseman, H. R. Brown, R. P. Sijbesma, C. Creton, Mechanics of elastomeric molecular composites. *Proc. Natl. Acad. Sci. U.S.A.* **115**, 9110–9115 (2018).

49. X. Li, J. P. Gong, Design principles for strong and tough hydrogels. *Nat. Rev. Mater.* **9**, 380–398 (2024).

50. J. P. Gong, Y. Katsuyama, T. Kurokawa, Y. Osada, Double-network hydrogels with extremely high mechanical strength. *Adv. Mater.* **15**, 1155–1158 (2003).

51. J. A. Greenwood, J. B. P. Williamson, Contact of nominally flat surfaces. *Proc. A* **295**, 300–319 (1966).

52. A. W. Bush, R. D. Gibson, T. R. Thomas, The elastic contact of a rough surface. *Wear* **35**, 87–111 (1975).

53. R. W. Smith, A. G. Veith, Electron microscopical examination of worn tire treads and tread debris. *Rubber Chem. Technol.* **55**, 469–482 (1982).

54. A. Schallamach, On the abrasion of rubber. *Proc. Phys. Soc. B* **67**, 883–891 (1954).

55. J. F. Archard, Elastic deformation and the laws of friction. *Proc. A* **243**, 190–205 (1957).

56. G. E. Sanoja, X. P. Morelle, J. Comtet, C. Joshua Yeh, M. Ciccotti, C. Creton, Why is mechanical fatigue different from toughness in elastomers? The role of damage by polymer chain scission. *Sci. Adv.* **7**, 1–23 (2021).

57. D. Zhao, A. Cartier, T. Narita, F. Lechenault, C. Creton, M. Ciccotti, Why cutting is easier than tearing elastomers. *Nat. Commun.* **16**, 3203 (2025).

58. X. P. Morelle, G. E. Sanoja, S. Castagnet, C. Creton, 3D fluorescent mapping of invisible molecular damage after cavitation in hydrogen exposed elastomers. *Soft Matter* **17**, 4266–4274 (2021).

59. R. Göstl, R. P. Sijbesma, Π-extended anthracenes as sensitive probes for mechanical stress. *Chem. Sci.* **7**, 370–375 (2016).

60. Z. J. Wang, S. Wang, J. Jiang, Y. Hu, T. Nakajima, S. Maeda, S. L. Craig, J. P. Gong, Effect of the activation force of mechanophore on its activation selectivity and efficiency in polymer networks. *J. Am. Chem. Soc.* **146**, 13336–13346 (2024).

61. J. Lengiewicz, M. de Souza, M. A. Lahmar, C. Courbon, D. Dalmas, S. Stupkiewicz, J. Scheibert, Finite deformations govern the anisotropic shear-induced area reduction of soft elastic contacts. *J. Mech. Phys. Solids* **143**, 104056 (2020).

62. R. Sahli, G. Pallares, A. Papangelo, M. Ciavarella, C. Ducottet, N. Ponthus, J. Scheibert, Shear-induced anisotropy in rough elastomer contact. *Phys. Rev. Lett.* **122**, 214301 (2019).

63. G. Carbone, C. Putignano, Rough viscoelastic sliding contact: Theory and experiments. *Phys. Rev. E* **89**, 1–9 (2014).

64. B. N. J. Persson, Theory of rubber friction and contact mechanics. *J. Chem. Phys.* **115**, 3840–3861 (2001).

65. R. A. Schapery, Analytical models for the deformation and adhesion components of rubber friction. *Tire Sci. Technol.* **6**, 3–47 (1978).

66. M. A. Miner, Cumulative damage in fatigue. *J. Appl. Mech.* **12**, A159–A164 (1945).

67. Q. Li, T. E. Tullis, D. Goldsby, R. W. Carpick, Frictional ageing from interfacial bonding and the origins of rate and state friction. *Nature* **480**, 233–236 (2011).

68. L. Bocquet, E. Charlaix, S. Ciliberto, J. Crassous, Moisture-induced ageing in granular media and the kinetics of capillary condensation. *Nature* **396**, 735–737 (1998).

69. D. Shohat, Y. Friedman, Y. Lahini, Logarithmic aging via instability cascades in disordered systems. *Nat. Phys.* **19**, 1890–1895 (2023).

70. H. Spikes, Stress-augmented thermal activation: Tribology feels the force. *Friction* **6**, 1–31 (2018).

71. B. N. J. Persson, Contact mechanics for randomly rough surfaces. *Surf. Sci. Rep.* **61**, 201–227 (2006).

72. G. Wu, P. Sotta, M. Huang, L. B. Tunnicliffe, J. J. C. Busfield, Characterization of sticky debris generated during smear wear. *Rubber Chem. Technol.* **96**, 588–607 (2023).

73. A. Ahagon, Chemical aspect of rubber abrasion. *Nippon Gomu Kyokaishi* **79**, 500–506 (2006).

74. E. Rabinowicz, The effect of size on the looseness of wear fragments. *Wear* **2**, 4–8 (1958).

75. J. Ju, G. E. Sanoja, L. Cipelletti, M. Ciccotti, B. Zhu, T. Narita, C. Y. Hui, C. Creton, Role of molecular damage in crack initiation mechanisms of tough elastomers. *Proc. Natl. Acad. Sci. U.S.A.* **121**, e2410515121 (2024).

76. H. W. Greensmith, Rupture of rubber. X. The change in stored energy on making a small cut in a test piece held in simple extension. *J. Appl. Polym. Sci.* **7**, 993–1002 (1963).

77. J. Ju, G. E. Sanoja, M. Y. Nagazi, L. Cipelletti, Z. Liu, C. Y. Hui, M. Ciccotti, T. Narita, C. Creton, Real-time early detection of crack propagation precursors in delayed fracture of soft elastomers. *Phys. Rev. X* **13**, 021030 (2023).

78. H. Liang, Y. Fukahori, A. G. Thomas, J. J. C. Busfield, The steady state abrasion of rubber: Why are the weakest rubber compounds so good in abrasion? *Wear* **268** 756–762 (2010).

79. A. Diaspro, F. Federici, M. Robello, Influence of refractive-index mismatch in high-resolution three-dimensional confocal microscopy. *Appl. Optics* **41**, 685–690 (2002).

80. T. D. B. Jacobs, T. Junge, L. Pastewka, Quantitative characterization of surface topography using spectral analysis. *Surf. Topogr. Metrol. Prop.* **5**, 013001 (2017).

81. B. N. J. Persson, O. Albohr, U. Tartaglino, A. I. Volokitin, E. Tosatti, On the nature of surface roughness with application to contact mechanics, sealing, rubber friction and adhesion. *J. Phys. Condens. Matter* **17**, R1–R62 (2005).

82. K. A. Grosch, F. P. Bowden, The relation between the friction and visco-elastic properties of rubber. *Proc. A* **274** 21–39 (1963).

83. D. Ahn, K. R. Shull, JKR studies of acrylic elastomer adhesion to glassy polymer substrates. *Macromolecules* **29** 4381–4390 (1996).

84. C. Creton, L. Leibler, How does tack depend on time of contact and contact pressure. *J. Polym. Sci. B* **34** 545–554 (1996).

85. L. Lomba, B. Giner, C. Lafuente, S. Martín, H. Artigas, Thermophysical properties of three compounds from the acrylate family. *J. Chem. Eng. Data* **58**, 1193–1202 (2013).

86. L. Andreozzi, V. Castelvetro, M. Faetti, M. Giordano, F. Zulli, Rheological and thermal properties of narrow distribution poly(ethyl acrylate)s. *Macromolecules* **39**, 1880–1889 (2006).
